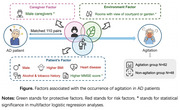# Exploring Modifiable Factors Influencing Agitation in Alzheimer's Disease: A Comprehensive Assessment

**DOI:** 10.1002/alz70860_100667

**Published:** 2025-12-23

**Authors:** Xinyi Qian, Bo Hong, Shifu Xiao, Xia Li, Ling Yue

**Affiliations:** ^1^ Shanghai Mental Health Center, Shanghai Jiao Tong University School of Medicine, Shanghai, China; ^2^ Alzheimer's Disease and Related Disorders Center, Shanghai Jiao Tong University, Shanghai, China; ^3^ Alzheimer's Disease and Related Disorders Center, Shanghai Jiao Tong University, Shanghai, Shanghai, China

## Abstract

**Background:**

This study aimed to comprehensively assess the impact of caregiver, environmental, and individual factors on agitation symptoms in Alzheimer's disease (AD) patients, identifying key modifiable factors.

**Method:**

From October 2022 to June 2023, 220 participants (110 AD patients and their primary caregivers) were recruited from Shanghai Mental Health Center. AD patients completed clinical surveys, including demographic, lifestyle and medical history information, and neuropsychological tests, such as the MMSE and the Geriatric Depression Scale (GDS). The caregivers completed the Neuropsychiatric Inventory and an environmental factors questionnaire for their care recipients, along with the Hamilton Depression Scale and Hamilton Anxiety Scale, to assess their own emotional state. Agitation severity was assessed using the Cohen‐Mansfield Agitation Inventory. Differences between groups (agitation vs. non‐agitation) and further relationships between potential factors and agitation were examined.

**Result:**

Among 110 AD patients, 56.36% exhibited agitation. The agitation group had more male patients (43.55% vs. 20.83%, *p* = 0.012) and female caregivers (79.03% vs. 52.08%, *p* = 0.003), lacked courtyard/garden views (70.97% vs. 91.97%, *p* = 0.007), had lower MMSE (12.18±7.44 vs. 16.15±6.95, *p* = 0.005) and GDS scores (4.00 vs. 6.00, *p* = 0.012). After adjusting variables, rooms with view of courtyard or garden (OR=0.218, 95% CI: 0.058–0.814; *p* = 0.023), male caregivers (OR=0.235, 95% CI: 0.087–0.630; *p* = 0.004), and high MMSE scores (OR=0.197, 95% CI: 0.066–0.586; *p* = 0.003) were identified as protective factors for agitation in AD patients.

**Conclusion:**

Our study demonstrated that agitation in AD patients can be improved by improving patients’ living environments, encouraging men to take on caregiver roles, and increasing caregiver support. Multifactorial intervention strategies involving physicians, communities, and families working together were advocated.